# Anti-Apoptotic and Pro-Survival Effect of *Alpinate Oxyphyllae Fructus* (AOF) in a d-Galactose-Induced Aging Heart

**DOI:** 10.3390/ijms17040466

**Published:** 2016-03-29

**Authors:** Yung-Ming Chang, Hen-Hong Chang, Wei-Wen Kuo, Hung-Jen Lin, Yu-Lan Yeh, Vijaya Padma Viswanadha, Chin-Chuan Tsai, Ray-Jade Chen, Hsin-Nung Chang, Chih-Yang Huang

**Affiliations:** 1 The School of Chinese Medicine for Post-Baccalaureate, I-Shou University, Kaohsiung 84001, Taiwan; dnas5728@yahoo.com.tw (Y.-M.C.); gimmy09200430@yahoo.com.tw (C.-C.T.); 2 Chinese Medicine Department, E-DA Hospital, Kaohsiung 82445, Taiwan; 3 1PT Biotechnology Co., Ltd., Taichung 433, Taiwan; 4 Research Center for Chinese Medicine & Acupuncture, China Medical University, Taichung 40402, Taiwan; glactose@hotmail.com; 5 Departments of Chinese Medicine, China Medical University Hospital, Taichung 40447, Taiwan; kawabunga0809@yahoo.com.tw; 6 School of Post-Baccalaureate Chinese Medicine, College of Chinese Medicine, China Medical University, Taichung 40402, Taiwan; 7 Department of Biological Science and Technology, China Medical University, Taichung 40447, Taiwan; wwkuo@mail.cmu.edu.tw; 8 Department of pathology, Changhua Christian Hospital, Changhua 50506, Taiwan; 1867@cch.org.tw; 9 Department of Medical Technology, Jen-Teh Junior College of Medicine, Nursing and Management, Miaoli 35665, Taiwan; 10 Department of Biotechnology, Bharathiar University, Coimbatore 641046, India; padma.vijaya@gmail.com; 11 Department of Surgery, School of Medicine, College of Medicine, Taipei Medical University, Taipei 11042, Taiwan; rayjchen@tmu.edu.tw; 12 Graduate Institute of Basic Medical Science, China Medical University, Taichung 40402, Taiwan; dadalidada0809@gmail.com; 13 School of Chinese Medicine, China Medical University, Taichung 40447, Taiwan; 14 Department of Health and Nutrition Biotechnology, Asia University, Taichung 41354, Taiwan

**Keywords:** AOF, d-galactose-induced aging, apoptosis, SIRT1

## Abstract

Aging, a natural biological/physiological phenomenon, is accelerated by reactive oxygen species (ROS) accumulation and identified by a progressive decrease in physiological function. Several studies have shown a positive relationship between aging and chronic heart failure (HF). Cardiac apoptosis was found in age-related diseases. We used a traditional Chinese medicine, *Alpinate Oxyphyllae Fructus* (AOF), to evaluate its effect on cardiac anti-apoptosis and pro-survival. Male eight-week-old Sprague–Dawley (SD) rats were segregated into five groups: normal control group (NC), d-Galactose-Induced aging group (Aging), and AOF of 50 (AL (AOF low)), 100 (AM (AOF medium)), 150 (AH (AOF high)) mg/kg/day. After eight weeks, hearts were measured by an Hematoxylin–Eosin (H&E) stain, Terminal deoxynucleotidyl transferase dUTP nick end labeling (TUNEL)-assays and Western blotting. The experimental results show that the cardiomyocyte apoptotic pathway protein expression increased in the d-Galactose-Induced aging groups, with dose-dependent inhibition in the AOF treatment group (AL, AM, and AH). Moreover, the expression of the pro-survival p-Akt (protein kinase B (Akt)), Bcl-2 (B-cell lymphoma 2), anti-apoptotic protein (Bcl-xL) protein decreased significantly in the d-Galactose-induced aging group, with increased performance in the AOF treatment group with levels of p-IGFIR and p-PI3K (Phosphatidylinositol-3′ kinase (PI3K)) to increase by dosage and compensatory performance. On the other hand, the protein of the Sirtuin 1 (SIRT1) pathway expression decreased in the aging groups and showed improvement in the AOF treatment group. Our results suggest that AOF strongly works against ROS-induced aging heart problems.

## 1. Introduction

The world’s elderly population is growing rapidly and a World Health Organization (WHO) report indicates that 22% of the world’s population will be aged over 60 years by 2050 [1]. The aging process progresses along an individual’s lifespan and is influenced by various pathological conditions such as cardiac disorders, diabetes mellitus, dementia, and neoplasia. Cardiovascular disease (CVD) is one of the major causes of death in this population [2,3,4,5]. CVDs are induced by various pathogenic factors such as lack of exercise, alcohol consumption, and smoking habits, especially in the elderly population [6,7,8].

Studies have demonstrated that oxidative stress increases with aging [9,10]. Moreover, some factors are highly associated with heart failure (HF) in aging, such as oxidative stress and inflammation [11].

Accumulating evidence has demonstrated that mitochondria are essential organelles with crucial functions in any tissue, such as energy metabolism and reactive oxygen species (ROS) generation [12]. Furthermore, heart failure is a serious cardiovascular disease associated with excess oxidative stress resulting from mitochondrial ROS accumulation [13,14,15]. Recently, a study indicated that ROS plays a critical role in heart failure, and under hypoxic conditions, mitochondria can overproduce ROS that may result in ROS-dependent hypoxia-induced cell death in cardiomyocytes [16].

The d-galactose-induced aged rat models have been widely used in studying aging mechanisms [17,18,19]. d-Galactose plays a role as a reducing sugar that reacts with amino groups in proteins, lipids, and nucleic acids to form advanced glycation endproducts (AGE) [20,21]. Formation and accumulation of AGEs increase ROS production by acting as the interacting receptor for AGE (RAGE) and accelerating the aging process [22,23,24,25,26]. Oxidative stress caused by ROS is considered a major factor leading to aberrant signaling pathways which finally contribute to the aging process [27,28,29].

Heart failure is a serious cardiovascular disease that impairs ventricle functions and contributes to cardiac multisystem disorders [30]. Myocardial apoptosis has been reported as an essential process in the development of HF [31,32,33,34]. Breaking the balance between cell death and cell survival mechanisms leads to heart failure [35].

Two main pathways—the “extrinsic” pathway and the “intrinsic pathway”—mediate apoptotic signaling in mammalian cells. The extrinsic apoptotic pathway is often triggered by P53 or Fas ligand which eventually activates the expression of death receptor superfamily members, such as Fas receptor and tumor necrosis factor-α receptor (TNFR) [34,36,37,38]. The death receptors induce the formation of a death-inducing signal complex (DISC) [39]. This complex recruits and aggregates the pro form of caspase 8 via the adaptor molecule Fas-associated death domain (FADD) [40], which leads to the activation of caspase 3, the key effector of apoptosis [41,42].

The intrinsic apoptotic pathway is also known as the mitochondria-dependent apoptotic pathway, mediated by Bax/Bcl-2 (B-cell lymphoma 2-associated X protein/B-cell lymphoma 2) dysregulation [43]. Intracellular signaling triggers outer mitochondrial membrane disruption, which would release cytochrome *c* from the mitochondria into the cytosol, which then triggers caspase 3 activation and results in apoptosis [36,43,44].

The anti-apoptotic protein Bcl2 inhibits the cytochrome *c* release from the mitochondria initiated by Bax [36]. Previous studies indicated that Bcl-2 overexpression in cardiomyocytes attenuates the release of mitochondrial inter-membrane proteins via a decrease in the loss of mitochondrial membrane electro-potential [45].

It is known that insulin and insulin-like growth factor-I (IGFI) signaling has important survival roles in cardiac tissues to promote the modulation of survival responses [46,47]. Phosphatidylinositol-3′ kinase (PI3K) and protein kinase B (Akt) have been identified as key determinants of insulin and IGFI receptor (IGFIR) signaling [48,49,50]. Previous studies indicated that IGFI signaling inactivated pro-apoptotic factor Bad through PI3K and the Akt pathway [51,52]. IGF1 signaling also promoted cardiac survival via activated increases in the anti-apoptotic protein (Bcl-xL) mitochondrial performance [53].

Sirtuin (SIRT) is a highly conserved family of class III histone deacetylases among species and widely expressed in almost all the mammalian organs. There are seven members (SIRT1-7) in the family. The sirtuin family plays an important role in many critical pathways, such as modulate stress-response and distinct metabolic pathways [54,55,56]. Sirtuin 1 (SIRT1), a nicotinamide adenine dinucleotide (NAD+)-dependent deacetylase, is involved in various cellular processes such as cell survival, apoptosis, growth, aging and metabolism [57,58,59]. Emerging evidence showed that SIRT1 is a longevity factor protecting cardiac myocytes against oxidative stress and attenuated cardiomyocyte hypertrophy and retards the progression of aging-induced cardiomyopathy [60,61].

*Alpinate Oxyphyllae Fructus* (*Alpinia oxyphylla* MIQ, AOF) is one of the important traditional Chinese medicines which has been widely used for treating salivation, polyuria, diarrhea, and gastralgia in light of the Chinese Pharmacopoeia [62]. Previous studies indicated that AOF extracts showed neuroprotective activity against oxidative stress-induced apoptosis [63]. AOF extracts also showed anti-apoptotic potential in cardio-myoblast cells. Our recent studies demonstrated that the Angiotensin-II induced cardiac apoptosis was significantly decreased by AOF extracts’ treatment [64]. In Korea, AOF was used for treating various symptoms accompanying hypertension and cerebrovascular disorders mainly because of its anti-aging and sexual-reinforcing activity [62,63,65,66,67]. Besides, it has been reported that the methanol extract of AOF has cardio-tonic effects [68]. Here, we investigated further whether AOF ameliorated the ROS-induced aging heart problem and related signaling paths and mechanisms.

## 2. Results

### 2.1. Echocardiography Findings

**Figure 1:**
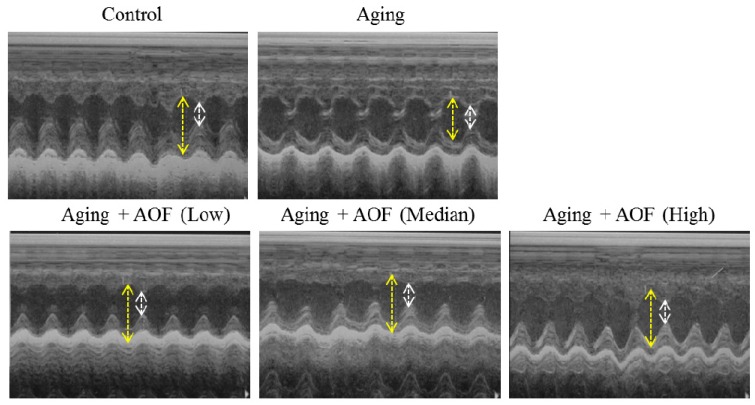
Echocardiography findings. Representative echocardiographic M-mode images from rats with d-galactose and *Alpinate Oxyphyllae Fructus* (AOF) treatment. AL (AOF low), AM (AOF medium), AH (AOF high) represent the doses of 50, 100 and 150 mg of *Alpinate Oxyphyllae Fructus* per kg BW (Body weight). The long yellow arrow indicates cardiac diastole, and the short white arrow shows cardiac systole.

We performed echocardiography to analyze heart function (Figure 1 and Table 1). We first examined whether d-galactose treatment for eight weeks induced rat cardiac aging. d-Galactose treatment significantly decreases heart function by FS% (fraction shortening (FS)) and EF% (ejection fraction (EF)) in the aging group rats (Figure 1). The echocardiographic parameters of Sprague–Dawley (SD) rats are presented in Table 1 with a significant difference in FS and EF between the aging group and AOF treatment group (Figure 1A). Eight weeks after being treated with low, median and high dosages of AOF, EF% were increased in these groups compared with the aging group (76.96 ± 2.86 *vs.* 67.46 ± 2.70, *p* < 0.01; 71.53 ± 0.77 *vs.* 67.46 ± 2.70, *p* < 0.05; and 71.53 ± 0.77 *vs.* 67.46 ± 2.70, *p* < 0.001, respectively). Additionally, we observed that the FS% was significantly increased compared with the aging group (41.12 ± 2.61 *vs.* 33.61 ± 2.02, *p* < 0.01; 36.52 ± 0.58 *vs.* 33.61 ± 2.02, *p* < 0.05; and 44.75 ± 3.92 *vs.* 33.61 ± 2.02, *p* < 0.001, respectively) (Figure 1B), thus indicating a cardioprotective effect.

### 2.2. Cardiac Histopathological Changes

**Figure 2:**
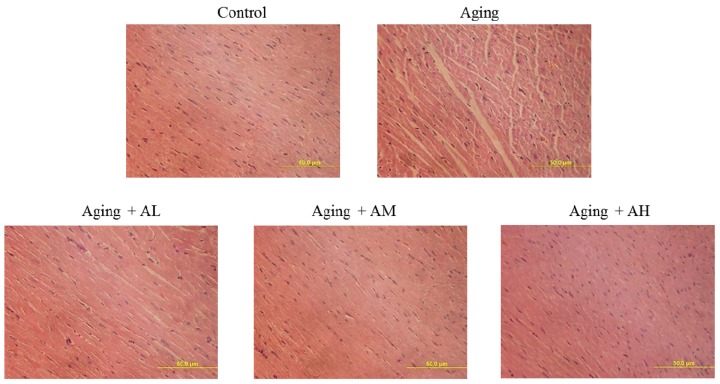
Morphological changes of rat cardiac tissue with Hematoxylin–Eosin (H&E) staining. Cardiac tissue sections stained with hematoxylin and eosin. The images of cardiac architecture were magnified ×400. The scale bar is 50 μm.

To investigate the changes in cardiac architecture, hematoxylin and eosin staining of tissue slides was performed to image cardiomyocytes (Figure 2). After viewing ×400 magnified images, the control group had normal myocardial cell architecture and volume. However, the aging groups exhibited abnormal myocardial architecture and volume due to aging. Cardiomyocytes from the aging rats were disordered with more space between the cells. However, the AOF treatment groups had significantly reduced disordered arrangement and space between cardiomyocytes (Figure 2).

### 2.3. TUNEL-Positive Cells Detection in Cardiac Tissues

**Figure 3:**
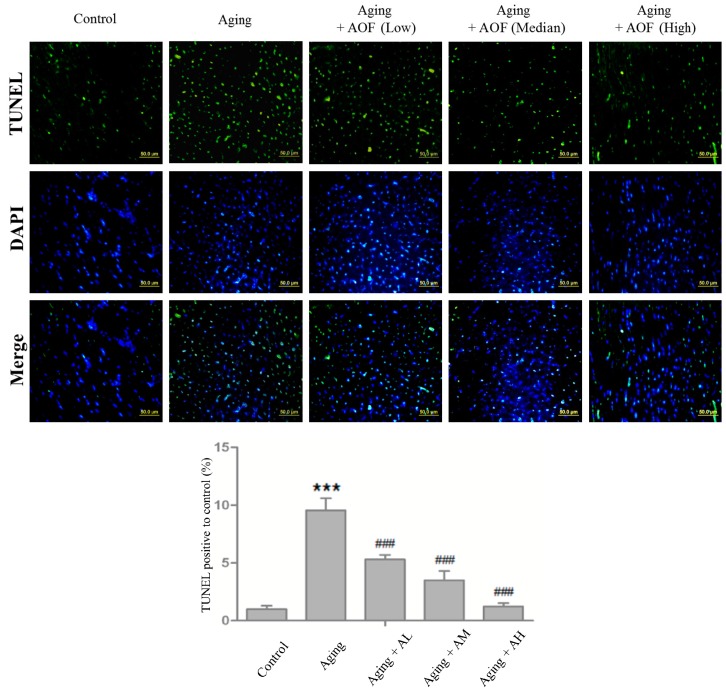
AOF treatment inhibits d-galactose-induced aging and apoptosis in Sprague–Dawley (SD) rat cardiac tissue. Cardiac tissue sections were stained with 4,6-diamidino-2-phenylindole (DAPI) (blue, nucleus) and TUNEL assay (green, double-stranded DNA break or single-stranded DNA nicks.), respectively. The statistical results were shown from three independent experiments; mean ± S.D.; *** *p* < 0.001, represent a significant difference *versus* the control; ^###^ *p* < 0.001, represent a significant difference *versus* the aging group.

Cell nuclei were stained with 4,6-diamidino-2-phenylindole (DAPI) (blue), and cleaved DNA fragments in the apoptotic nuclei were detected by terminal deoxynucleotidyl transferase dUTP nick end labeling (TUNEL) staining (green). The images were magnified ×200, TUNEL staining showed that the aging group rats had higher number of apoptotic cardiomyocytes compared to the control group. Additionally, the AOF treatment groups (AL, AM and AH) had fewer apoptotic cardiac cells than those in the aging group (Figure 3).

Moreover, the Image J software analyzed the number of apoptotic cells with one-way analysis of variance (ANOVA) statistical analysis, showing differences with *p* < 0.001 for Control:Aging and *p* < 0.001 for Aging:(Aging + AL/AM/AH). The results in Figure 3 show significant differences.

### 2.4. Alpinate Oxyphyllae Fructus (AOF) Treatment Attenuated the Activation of Cardiac Fas Receptor-Dependent Apoptotic Pathways

**Figure 4:**
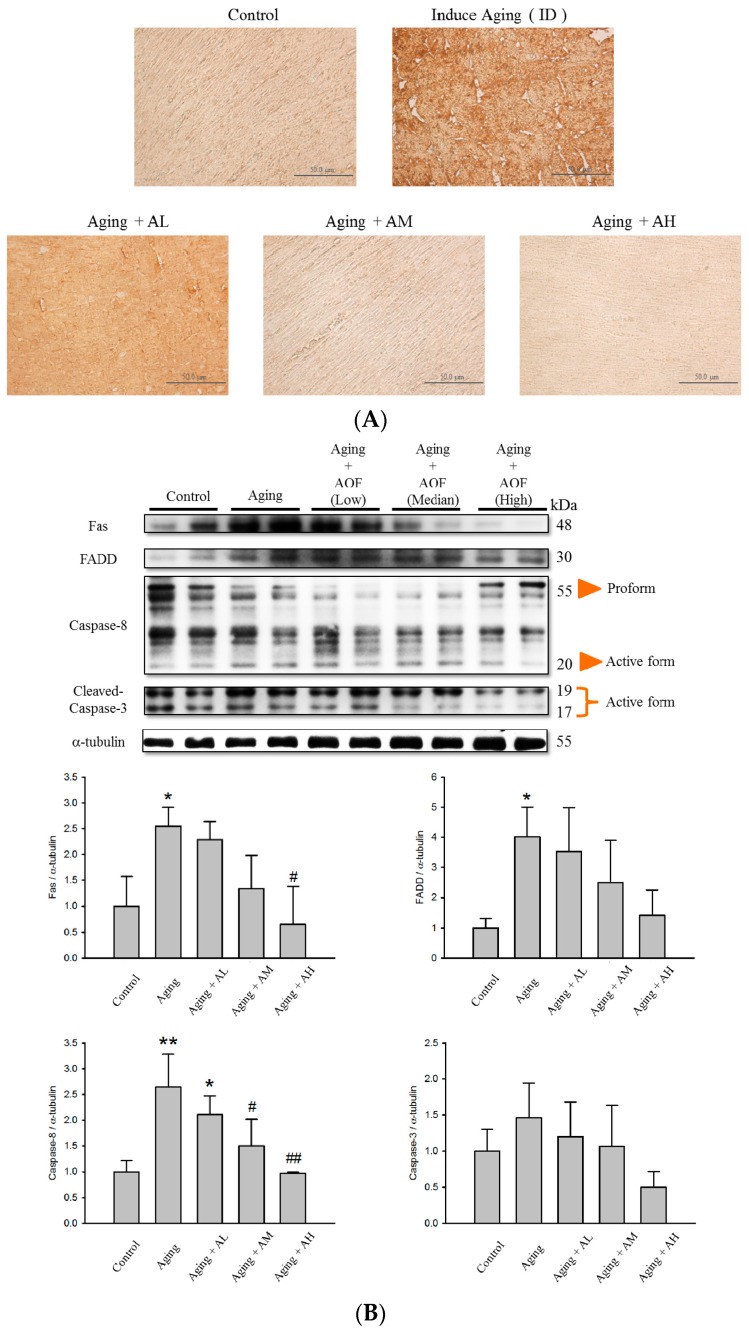

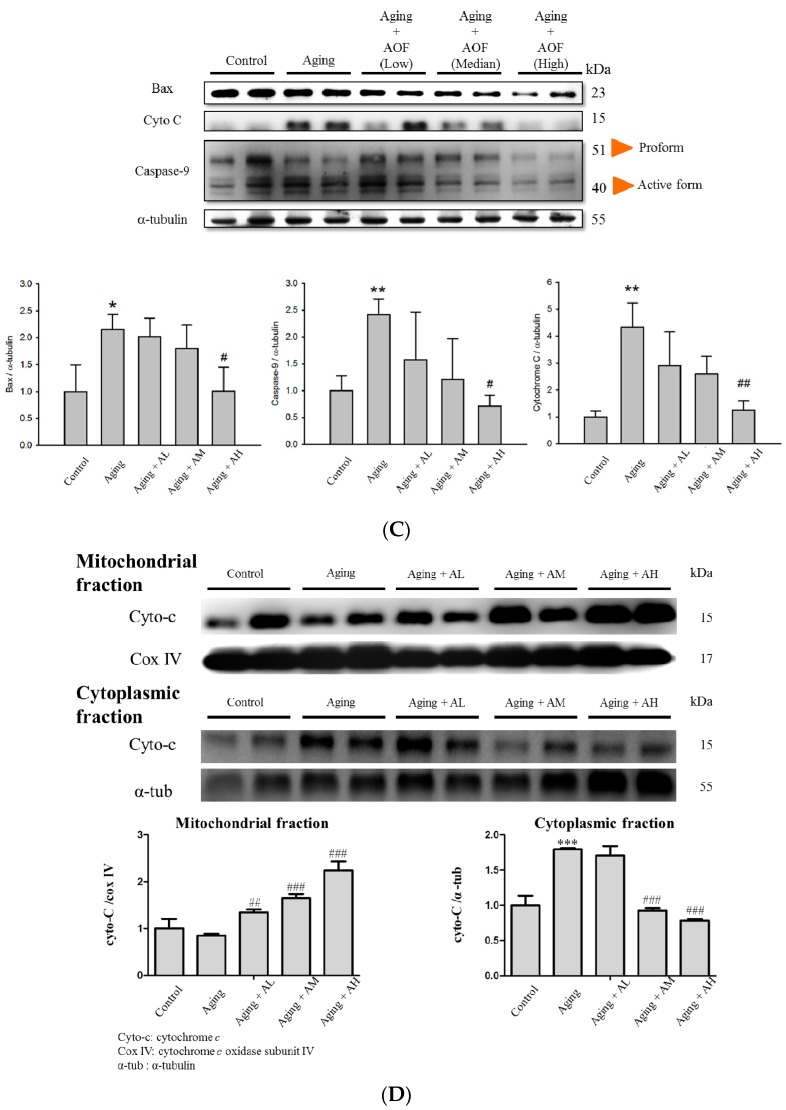
Effect of AOF on d-galactose -induced apoptosis in SD rat cardiac tissue. (**A**) Immunohistochemical analysis for cleaved Caspase-3 in sections from the SD rat cardiovascular tissue. The protein levels decreased with AOF treatment and are shown in a brown color. Final magnifications: ×400 (bar, 50 μm). The total protein of SD rat cardiac tissue extracts was separated by 12% SDS polyacrylamide gel electrophoresis (SDS-PAGE), transferred to polyvinylidene difluoride (PVDF) membranes, and immunoblotted with antibodies against Fas, Fas-associated death domain (FADD), caspase-8, cleaved caspase-3, B-cell lymphoma 2-associated X protein (Bax), cytochrome *c* and caspase-9 antibody to detect apoptotic markers expression (**B**,**C**); Levels of cytochrome *c* were determined from cytoplasmic and from the mitochondria (**D**). Equal loading was assessed with an anti-α-tubulin antibody. These blots were quantified by densitometry. α-tubulin served as a loading control. Data are presented as means ± S.D. Bars indicate averages, * *p* < 0.05; ** *p* < 0.01; *** *p* < 0.001, represent a significant difference *versus* the control; ^#^ *p* < 0.05; ^##^ *p* < 0.01; ^###^ *p* < 0.001, represent a significant difference *versus* the aging group. *n* = three independent experiments for each data point.

To investigate whether AOF could inhibit cardiac cell apoptosis in d-galactose-induced aging rat models, the protein level of cleaved Caspase-3 in the tissue sections were examined by immunohistochemistry (IHC). The result shows cleaved Caspase-3 staining was stronger in the aging group compared to normal tissue. Moreover, representative imaging demonstrated that treatment with AOF significantly decreased the protein level of cleaved Caspase-3 in the d-galactose-induced aging rats models (Figure 4A).

After confirming the aging effects of the d-galactose treatment, we investigated how mitochondria and caspase dependent apoptotic signaling pathways were altered in aging rats that were fed with AOF. The Western blot result showed that the d-galactose treatment induced Caspase 3, 8, 9, Bax levels (Figure 4B,C) and Cytochrome *c* release in the cytosol (Figure 4D). However, all these changes induced by d-galactose were totally reversed by AOF in a dose-dependent manner (Figure 4). All the data suggest that AOF may have a strong cardio-protective function by decreasing the apoptotic pathway.

### 2.5. Effect of AOF Treatment on Cardiac Survival Pathways

**Figure 5:**
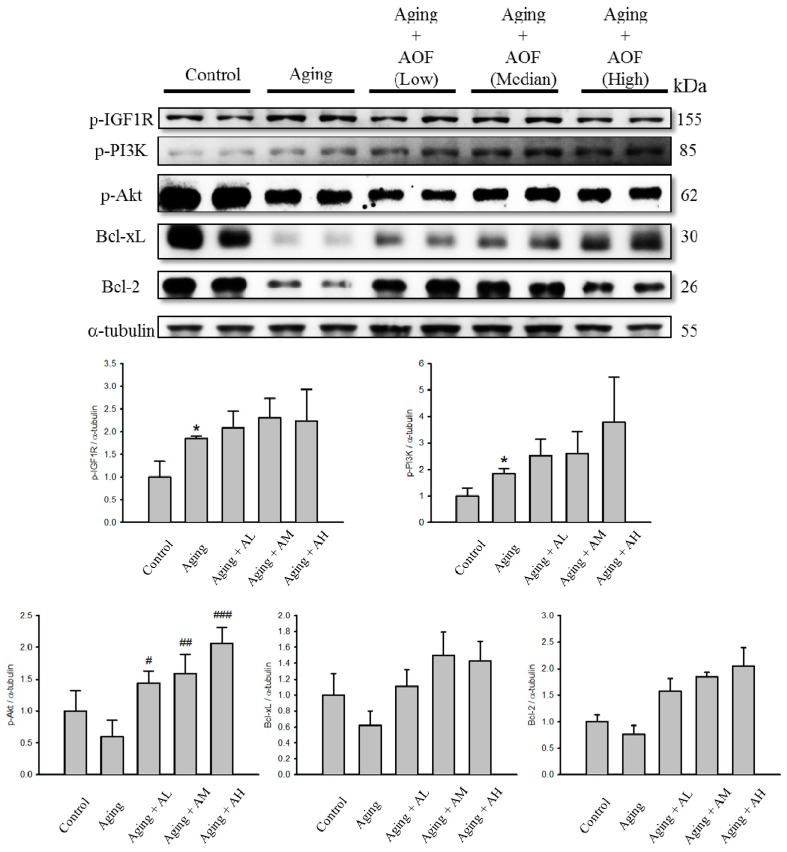
Effect of AOF on d-galactose-induced survival in SD rat cardiac tissue. The total protein of SD rat cardiac tissue extracts was separated by 12% SDS-PAGE, transferred to PVDF membranes, and immunoblotted with antibodies against IGF1R, p-PI3K, AKT, Bcl-xL and Bcl2 antibody to detect survival markers expression. Equal loading was assessed with an anti-α-tubulin antibody. These blots were quantified by densitometry. α-tubulin served as a loading control. Data are presented as means ± S.D. Bars indicate averages, * *p* < 0.05, represent a significant difference *versus* the control; ^#^ *p* < 0.05; ^##^ *p* < 0.01; ^###^ *p* < 0.001, represent a significant difference *versus* the aging group. *n* = three independent experiments for each data point.

We further examined whether the survival proteins’ expression increased with AOF treatment in the aging rat hearts. The protein levels of p-Akt, Bcl-2, Bcl-xL, p-IGF1R and p-PI3K were significantly decreased in the aging group, but after being treated with AOF, the survival protein level increased significantly compared with those in the aging group (Figure 5).

**Figure 6:**
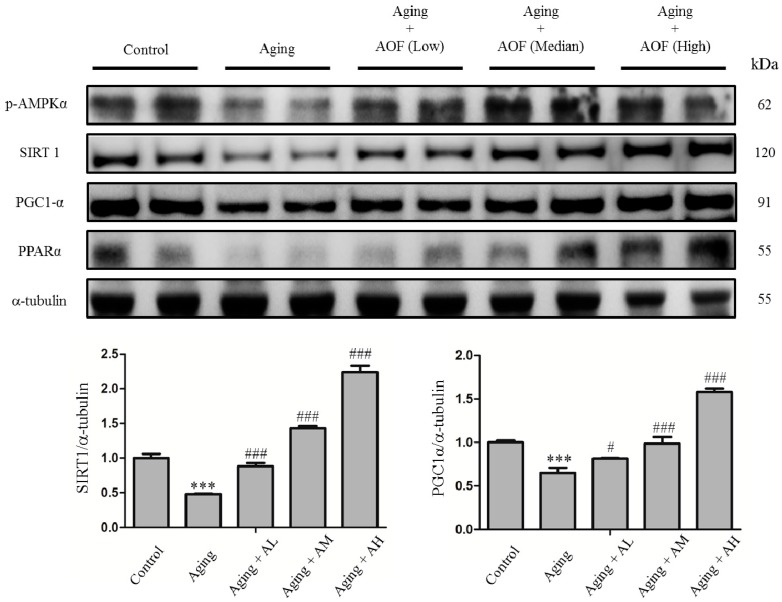
Effect of AOF on d-galactose-induced Sirtuin 1 (SIRT1) pathway related protein in SD rat cardiac tissue. The total protein of SD rat cardiac tissue extracts was separated by 12% SDS-PAGE, transferred to PVDF membranes, and immunoblotted with antibodies against p-AMPK, SIRT1, PGC-1α and PPARα antibody to detect survival markers expression. Equal loading was assessed with an anti-α-tubulin antibody. These blots were quantified by densitometry. α-tubulin served as a loading control. Data are presented as means ± S.D. Bars indicate averages, *** *p* < 0.001, represent a significant difference *versus* the control; ^#^ *p* < 0.05; ^###^ *p* < 0.001, represent a significant difference *versus* the aging group. *n* = three independent experiments for each data point.

According to our previous study, aging rats performed the SIRT longevity pathway instead of the IGF1 survival signaling to increase cardiomyocyte survival [69]. Our study also focused on the longevity-related signaling molecules, phospho AMP-activated protein kinase (p-AMPK), SIRT1, and peroxisome proliferator-activated receptor-γ co-activator-1 α (PGC-1α) (Figure 6). The SIRT1 pathway protein decreased with age. The protein levels of p-AMPK, SIRT1, and PGC-1α were significantly lower with age in the aging groups. Comparing the treatment of the AOF groups (AL, AM, and AH) to aging groups, in which p-AMPK, SIRT1, and PGC-1α were higher, a dose-dependent manner was demonstrated in this longevity-related signaling pathway.

## 3. Discussion

The results from our study show that d-galactose induced aging in rats incurred cardiomyocyte apoptosis. However, eight weeks of AOF treatments provided remarkable benefits in d-galactose induced aging rats. In addition, AOF treatment enhanced the protein levels of p-Akt, Bcl-2 and Bcl-xL in cardiomyocytes, and decreased the levels of Caspase 3, 8, 9, Bax and cytosolic Cytochrome *c* and thereby inhibited cellular apoptosis. These results demonstrated that treatment with AOF efficiently attenuates cardiomyocyte apoptosis of aging rats.

We used a traditional Chinese medicine *Alpinate Oxyphyllae Fructus*, which has been reported with neuroprotective activity and anti-apoptotic potential in cardiomyoblast cells [62,64,65]. In our previous experiment, we found that poor health factors can cause heart disease, such as high blood pressure, obesity, diabetes, and even secondhand smoke in rats. The apoptotic pathway exhibited increased levels of damage with a reduced survival pathway in these situations. Exercise training and eating purple sweet potato yogurt can help to prevent heart failure and apoptosis [70,71,72,73,74]. Few studies have investigated whether AOF can prevent cardiac apoptosis in aging.

In cardiomyocytes, mitochondria perform a dual role in continuous supply of ATP providing the contracting cardiacmyocyte and cell apoptosis. In response to changes in the environment, mitochondria quickly change from energy supplier to cell death promoter. Mitochondrial dysfunction leads to ATP synthesis disruption and further produce ROS, finally resulting in cardiacmyocyte apoptosis [75,76,77]. It has been reported that ROS contributes to the development of heart failure as it correlates with left ventricle (LV) dysfunction [78,79]. Our findings showed that the d-galactose stimuli ROS accumulation resulted in cardiomyocytes disorder and significantly decreased heart function on FS% and EF%. However, previous studies have found AOF to be beneficial in cardiac survival [64]. Thus, we expected AOF may therefore demonstrate recovery potential for cardiac cell morphology. As expected, heart function and cardiomyocyte disorder were significantly rescued by AOF treatment. In terms of functional assay, AOF treatment could provide benefits in cardiomyocytes.

d-Galactose-induced aging rats exhibit many symptoms similar to natural aging, such as poor immune responses, decreased antioxidant enzyme activity and accumulation of ROS [80,81,82]. Galactose metabolism is divided into three major metabolic pathways. However, one of these pathways, galactose-oxidase trigger galacitol (ducitol) and O_2_ to aldehydes and H_2_O_2_ major dominate ROS accumulation [83]. The levels of reducing sugars were significantly increased in d-galactose-induced aging mice which led to excessive galactose metabolism. Many studies have demonstrated that d-galactose induced aging symptoms are due to the oxidative stress resulting from excessive galactose metabolism [18,84,85]. The superoxide content has also been found to have increased dramatically in d-galactose-induced aging mice brains and livers [86].

Apoptosis is a critical event often associated with the pathophysiology of heart failure [87,88,89]. We hypothesized that the d-galactose-induced cell apoptosis could be recovered in our d-galactose-induced aged rat. In the present study, d-galactose increased cardiomyocytes apoptosis levels, as indicated with the increase in TUNEL-positive cells. However, this situation was reversed by AOF treatment (50, 100, and 150 mg/Kg/day), leading to an apoptotic level similar to that of the control (Figure 3). The activation of Caspase-9 and -3 also increased by d-galactose-induced aging and down-regulated by AOF administration (Figure 4). The level of anti-apoptotic proteins were reduced in the d-galactose-induced aging group, but were reversed by AOF treatment (Figure 5).

In addition, growing evidence supports a close relationship between inflammation and oxidation [90]. ROS overproduction triggers the tumor necrosis factor-α (TNF-α) and nuclear transcription factor-κB (NF-κB) related inflammatory signaling in cardiomyocytes [91]. TNF-α enhanced by ROS further activates NF-κB, which also mediates formation of a death-inducing signal complex (DISC) by TNFα receptor, eventually leading to the activation of extrinsic apoptotic pathway [40,92,93]. NF-κB had been characterized as a central mediator of inflammatory responses and is involved in the regulation of cellular apoptosis [94,95,96]. In pro-inflammatory myocardium, ROS triggers the toll-like receptors (TLR) involved in intracellular signaling and activates NF-κB by proteasomal degradation of the inhibitors of NF-κB (IκBs), which result in the nuclear translocation of NF-κB and further expression of pro-inflammatory cytokines such as interleukin (IL)-1β, IL-18 and activate NLRP3 (NLR Family, Pyrin Domain Containing 3) inflammasome [97]. The NLRP3-inflammasome is composed of procaspase-1, NLRP3 and adapter protein apoptosis-associated speck-like protein (ASC) [98,99]. Inflammasome effector caspase-1 eventually processes IL-1β and IL-18 precursors to their active forms and further triggers multiple pro-inflammatory pathways [100,101].

Emerging evidence indicates that NF-κB and SIRT1 signaling are antagonistic mechanisms that maintain cellular homeostasis [95]. SIRT1 triggers the downstream effects of AMPK, PGC-1α and peroxisome proliferator-activated receptor-α (PPAR-α). These factors activate oxidative metabolism and suppress both NF-κB signaling and inflammation. On the other hand, the NF-κB system also down-regulates SIRT1-mediated function via the miR-34a expression and reactive oxygen species [95,102]. SIRT1 inhibits NF-κB transcriptional activity by directly interacting with the NF-κB subunit RelA/p65 and deacetylating RelA/p65 at lysine 310 [103]. To date, most studies suggested that SIRT1 mediates PPARs’ activation by PGC1α deacetylation, ensuring PPARα/PGC-1α down-regulates TLR and inflammasome-dependent inflammation, respectively, by inhibition of p38 MAPK and inflammasome assembly [104,105,106,107,108,109]. Our data demonstrate that SIRT was markedly decreased in rat hearts with d-galactose treatment. However, the positive effects resulting from the AOF treatment include activation of longevity factor SIRT 1 and promotion of the SIRT1-mediated functions through PPARα/PGC-1α activation (Figure 6). Therefore, AOF could effectively restrain myocardial apoptosis in aging rats.

The cardio-protective ability of sulphonylurea receptor subunits SUR2A—an “atypical” ATP-binding cassette (ABC) protein—has been previously revealed in aging rats [110]. Overexpression of myocardial SUR2A has been suggested to increase cell resistance against metabolic stress and aging-induced decline in cardiac health [110,111,112]. Interestingly, emerging evidence indicates that the PI3K/Akt signaling pathway is important for up-regulation of SUR2A [113,114]. Furthermore, recent study indicates that high NAD+/NADH ratio up-regulates SUR2A expression via PI3K/Akt signaling and increases cardiac resistance to different types of stresses [113,115,116,117]. NAD^+^/NADH ratio plays an important role in DNA repair, cell death, oxidative metabolism, and ageing process [118]. It is known that sirtuins process deacylation reactions uniquely with the co-substrate NAD^+^ and are been described as sensors of the NAD^+^/NADH ratio [119]. Subsequent studies demonstrate up-regulation of SIRT1 and activation of oxidative metabolism when the ratio moves towards higher NAD^+^ [120,121]. Correlation of these signaling factors with SIRT1 and SUR2A suggest that AOF treatment might also increase SUR2A levels. However, whether SUR2A is involved in the positive effect of AOF and their associated mechanisms remains unknown.

In conclusion, it was found that aging induced significant increases of apoptosis in cardiomyocytes. However, AOF reduced these effects in treatment by different dosages. Therefore, AOF might be effective for cardiac apoptosis and ventricular remodeling prevention in aging-enhanced cardiovascular diseases.

## 4. Materials and Methods

### 4.1. AOF Extraction

Fragments of AOF were obtained from Shin-Long Pharmaceutical Company (Taichung, Taiwan). The AOF fragments (150 g) were extracted with 600 mL of boiling distilled water for 2 h. The AOF filtrate was concentrated under reduced pressure and then stored at 4 °C for further use. The spray drying was used to produce AOF extract powder.

### 4.2. Animals and Experimental Design

Thirty-two male 8-week-old Sprague–Dawley (SD) rats weighing approximately 220 ± 20 g were used in the research. Animals were purchased from BioLASCO Taiwan Co., Ltd., (Taipei, Taiwan) and cared for at the University Animal center, China Medical University in accordance with Institutional Animal Care and Use Committee regulations. Rats were kept in a temperature-controlled (23 ± 2 °C) room with a 12:12-h light–dark cycle, and water and rat chow were provided *ad libitum*. After a 2-week acclimation period, the rats were randomly divided into five groups and named as control group, aging group (which was intraperitoneal (IP) injection injected with 150 mg/kg/day of d-galactose for 8 weeks), AOF low (AL, aging rats with 50 mg/kg/day of AOF), AOF medium (AM, aging rats with 100 mg/kg/day of AOF), AOF high (AH, aging rats with 150 mg/kg/day of AOF). The AOF was administered to the AOF group using oral gavage and the other groups were given the same volume of control solution. Rats were sacrificed at the end of the treatment, and heart tissue was immediately collected or stored at −80 °C until further use. All experimental procedures were following the National Institutes of Health (NIH) Guide for the Care and Use of Laboratory Animals. The animal use experimental protocol was approved by the Institutional Animal Care and Use Committee (IACUC) of China Medical University, Taichung, Taiwan (No.102-71-N; 15 August 2013).

### 4.3. Echocardiography

Several heart functions were examined by echocardiography including left ventricular internal end-diastolic dimensions (LVIDd), left ventricular internal end-systolic dimensions (LVIDs), inter-ventricular septum (IVS), posterior wall thicknesses (LVPW), end diastolic velocity (EDV), end systolic velocity (ESV) and fractional shortening (FS) and ejection fraction (EF). FS% was calculated according to the following equation: FS% = ((LVIDd − LVIDs)/LVIDd) × 100, and EF% was calculated according to the following equation: EF (Teich) [%] = [(EDV − ESV)/EDV] × 100.

### 4.4. Hematoxylin–Eosin (H & E) Staining

The tissue sections were dyed using hematoxylin and eosin (H & E). Sections were deparaffinized by immersion in xylene and dyed using hematoxylin for 3 min. Sections were washed three times in double-distilled water (DDW) and then placed in 85% alcohol for 2 min. Then, the sections were dyed with eosin for 5 min and dehydrated through graded alcohols (90%, 80% and 70%). Finally, heart tissues were soaked in xylene, dried and morphological changes in the stained sections were examined under light microscopy (OLYMPUS Microscope, Tokyo, Japan).

### 4.5. Immunohistochemistry

Four micrometer thick paraffin sections were deparaffinized in xylene and sequentially rehydrated using a graded series of ethanol. The endogenous peroxidase activity was blocked with 3% hydrogen peroxide. After rinsing in water for 15 min, the sections were microwave-treated with pre-warmed citrate buffer (10 mM citric acid, pH 6.0) for 15 min, cooled down to room temperature (RT) for 30 min, and blocked with 5% cosmic calf serum (CCS, HyClone, UT, USA) for 1 h. The sections were incubated with cleaved Caspase-3 antibody (1:100) overnight at 4 °C. Then, the sections were incubated with the appropriate secondary antibodies (Santa Cruz Biotechnology, Dallas, TX, USA) for 15 min at RT. Immunoreactivity was detected with 3,3′-diaminobenzidine (DAB) substrate (Roche, Mannheim, Germany) for 5 min and the samples were washed with 1× phosphate-buffered saline (PBS, Gibco, Grand island, NY, USA) for 10 min. The sections were then viewed by using microscopy (magnification: ×200) (OLYMPUS Microscope, Tokyo, Japan).

### 4.6. 4,6-Diamidino-2-phenylindole (DAPI) and Tunnel Staining

The cardiac sections were incubated with proteinase K (20 µg/mL) and the washed in phosphate-buffered saline. The sections were then incubated with terminal deoxynucleotidyl transferase and fluorescein isothiocyanate-dUTP (TUNEL, Roche Applied Science, Indianapolis, IN, USA) for 60 min at 37 °C. TUNEL-positive nuclei (fragmented DNA) were appeared as bright green spots at 460 nm. The 4,6-diamidino-2-phenylindole (DAPI) stain was dissolved in PBS at 0.1 μg/mL and applied on to the slides and incubated for 5 min and the nuclei were stained in blue light at 454 nm. Photomicrographs were recorded using a Zeiss Axiophot microscope (Zeiss Axiophot, Oberkochen, Deutschland, Germany). The counts were made by at least two different individuals in a blinded manner.

### 4.7. Tissue Extraction

The left ventricle tissues extracts were obtained by homogenizing in a lysis buffer (50 mM Tris-HCL, pH 7.4, 2 mM EDTA, 50 mM NaF, 150 mM NaCl, 1% NP-40, 0.5% Na-deoxycholate, 0.1% SDS) at a ratio of 100 mg tissue/mL buffer for 2 min. The homogenates were placed on ice for 10 min and then centrifuged twice at 12,000× *g* for 40 min. The clean upper layer suspension was collected and stored at −80 °C for further experiments.

### 4.8. Electrophoresis and Western Blot

The protein concentration of cardiac tissue extracts were determined by the Lowry protein assay. The samples (40 µg/lane) were separated by 10% SDS polyacrylamide gel electrophoresis (SDS-PAGE). Proteins were then transferred to polyvinylidene difluoride (PVDF) membrane (Millipore, Bedford, MA, USA). The membranes were blocked in 5% milk in TBS buffer for 2 h with rotation. After washed with TBS buffer 3 times, membranes were incubated overnight at 4 °C with primary antibody. The immunoblots were washed with TBS buffer 3 times for 10 min each and then incubated with the secondary antibody for 1 h at room temperature. The signals were visualized with an enhanced chemiluminescence (ECL) reagent (Santa Cruz Biotechnology, Santa Cruz, CA, USA). Relative density of the blots was quantified using Image J software (NIH, Bethesda, MD, USA).

### 4.9. Statistical Analysis

All the experimental data are expressed as mean ± S.D. Comparison between two-groups were performed with Student’s *t*-tests; statistical comparisons between multiple groups were performed by one-way ANOVA. In all cases, a value of *p* < 0.05 was considered significant.

## Figures and Tables

**Table 1:** Echocardiographic parameters in the studied groups.

Echocardiographic Parameters	Control	Aging	Aging + AL	Aging + AM	Aging + AH
	*n* = 3	*n* = 3	*n* = 3	*n* = 3	*n* = 3
EF (Teich) (%)	75.88 ± 6.58	67.46 ± 2.70	76.96 ± 2.86 ^##^	71.53 ± 0.77 ^#^	80.56 ± 3.79 ^###^
FS (%)	40.44 ± 5.83	33.61 ± 2.02	41.12 ± 2.61 ^##^	36.52 ± 0.58	44.75 ± 3.92 ^##^

Data shown are means ± S.D. *n* = 3 at least in each group. AL, *Alpinate Oxyphyllae Fructus* (AOF) low; AM, AOF medium; AH, AOF high; FS, Fractional shortening; EF, ejection fraction. ^#^ *p* < 0.05; ^##^ *p* < 0.01; ^###^ *p* < 0.001 *vs.* Aging group, respectively.

## References

[1] Larbi A., Rymkiewicz P., Vasudev A., Low I., Shadan N.B., Mustafah S., Ayyadhury S., Fulop T. (2013). The immune system in the elderly: A fair fight against diseases?. Aging Health.

[2] Enns L.C., Wiley J.C., Ladiges W.C. (2008). Clinical relevance of transgenic mouse models for aging research. Crit. Rev. Eukaryot. Gene Expr..

[3] Zweier J.L., Talukder M.A. (2006). The role of oxidants and free radicals in reperfusion injury. Cardiovasc. Res..

[4] Sun S.L., Guo L., Ren Y.C., Wang B., Li R.H., Qi Y.S., Yu H., Chang N.D., Li M.H., Peng H.S. (2014). Anti-apoptosis effect of polysaccharide isolated from the seeds of Cuscuta chinensis Lam on cardiomyocytes in aging rats. Mol. Biol. Rep..

[5] Odden M.C., Shlipak M.G., Whitson H.E., Katz R., Kearney P.M., Defilippi C., Shastri S., Sarnak M.J., Siscovick D.S., Cushman M. (2014). Risk factors for cardiovascular disease across the spectrum of older age: The Cardiovascular Health Study. Atherosclerosis.

[6] Blondon M., Wiggins K.L., McKnight B., Psaty B.M., Rice K.M., Heckbert S.R., Smith N.L. (2013). The association of smoking with venous thrombosis in women. A population-based, case-control study. Thromb. Haemost..

[7] Fernhall B. (2013). Long-term aerobic exercise maintains peak VO_2_, improves quality of life, and reduces hospitalisations and mortality in patients with heart failure. J. Physiother..

[8] Gargiulo G., Testa G., Cacciatore F., Mazzella F., Galizia G., Della-Morte D., Langellotto A., Pirozzi G., Ferro G., Ferrara N. (2013). Moderate alcohol consumption predicts long-term mortality in elderly subjects with chronic heart failure. J. Nutr. Health Aging.

[9] Liu D., Xu Y. (2011). p53, oxidative stress, and aging. Antioxid. Redox Signal..

[10] Finkel T., Holbrook N.J. (2000). Oxidants, oxidative stress and the biology of ageing. Nature.

[11] Madamanchi N.R., Runge M.S. (2013). Redox signaling in cardiovascular health and disease. Free Radic. Biol. Med..

[12] Akhmedov A.T., Marin-Garcia J. (2015). Mitochondrial DNA maintenance: An appraisal. Mol. Cell. Biochem..

[13] Cadenas S., Aragones J., Landazuri M.O. (2010). Mitochondrial reprogramming through cardiac oxygen sensors in ischaemic heart disease. Cardiovasc. Res..

[14] Munzel T., Gori T., Keaney J.F., Maack C., Daiber A. (2015). Pathophysiological role of oxidative stress in systolic and diastolic heart failure and its therapeutic implications. Eur. Heart J..

[15] Hsieh S.R., Cheng W.C., Su Y.M., Chiu C.H., Liou Y.M. (2014). Molecular targets for anti-oxidative protection of green tea polyphenols against myocardial ischemic injury. BioMedicine.

[16] Kuo C.Y., Chiu Y.C., Lee A.Y., Hwang T.L. (2015). Mitochondrial Lon protease controls ROS-dependent apoptosis in cardiomyocyte under hypoxia. Mitochondrion.

[17] Zhang Z.F., Fan S.H., Zheng Y.L., Lu J., Wu D.M., Shan Q., Hu B. (2009). Purple sweet potato color attenuates oxidative stress and inflammatory response induced by d-galactose in mouse liver. Food Chem. Toxicol..

[18] Zhou Y.Y., Ji X.F., Fu J.P., Zhu X.J., Li R.H., Mu C.K., Wang C.L., Song W.W. (2015). Gene Transcriptional and metabolic profile changes in mimetic aging mice induced by d-galactose. PLoS ONE.

[19] Zhang X.L., Jiang B., Li Z.B., Hao S., An L.J. (2007). Catalpol ameliorates cognition deficits and attenuates oxidative damage in the brain of senescent mice induced by d-galactose. Pharmacol. Biochem. Behav..

[20] Bucala R., Cerami A. (1992). Advanced glycosylation: Chemistry, biology, and implications for diabetes and aging. Adv. Pharmacol..

[21] Vlassara H., Bucala R., Striker L. (1994). Pathogenic effects of advanced glycosylation: Biochemical, biologic, and clinical implications for diabetes and aging. Lab. Investig..

[22] Wang Z.H. (2014). Anti-glycative effects of asiatic acid in human keratinocyte cells. Biomedicine.

[23] Munch G., Westcott B., Menini T., Gugliucci A. (2012). Advanced glycation endproducts and their pathogenic roles in neurological disorders. Amino Acids.

[24] Calcutt N.A., Cooper M.E., Kern T.S., Schmidt A.M. (2009). Therapies for hyperglycaemia-induced diabetic complications: From animal models to clinical trials. Nat. Rev. Drug Discov..

[25] Song X., Bao M., Li D., Li Y.M. (1999). Advanced glycation in d-galactose induced mouse aging model. Mech. Ageing Dev..

[26] Zhang X., Jin C., Li Y., Guan S., Han F., Zhang S. (2013). Catalpol improves cholinergic function and reduces inflammatory cytokines in the senescent mice induced by d-galactose. Food Chem. Toxicol..

[27] Fujihara M., Azuma H., Ikeda H., Yamaguchi M., Hamada H. (2011). Bone marrow stromal cell line promotes the proliferation of mast cell progenitors derived from cord blood CD34^+^ cells under serum-free conditions with a combination of both cell–cell interaction and soluble factors. Artif. Cells Blood Substit. Immobil. Biotechnol..

[28] Rai P., Onder T.T., Young J.J., McFaline J.L., Pang B., Dedon P.C., Weinberg R.A. (2009). Continuous elimination of oxidized nucleotides is necessary to prevent rapid onset of cellular senescence. Proc. Natl. Acad. Sci. USA.

[29] Choi Y.J., Kim D.H., Lee E.K., Kim J.M., Ha Y.M., Kim N.D., Jung J.H., Choi J.S., Yu B.P., Chung H.Y. (2012). Attenuation of age-related changes in FOXO3a activity and the PI3K/Akt pathway by short-term feeding of ferulate. Age.

[30] Jessup M., Abraham W.T., Casey D.E., Feldman A.M., Francis G.S., Ganiats T.G., Konstam M.A., Mancini D.M., Rahko P.S., Silver M.A. (2009). 2009 Focused update: ACCF/AHA Guidelines for the diagnosis and management of heart failure in adults: A report of the American College of Cardiology Foundation/American Heart Association Task force on practice guidelines: Developed in collaboration with the International Society for Heart and Lung Transplantation. Circulation.

[31] Haunstetter A., Izumo S. (1998). Apoptosis: Basic mechanisms and implications for cardiovascular disease. Circ. Res..

[32] Ahn D.H., Singaravelu G., Lee S., Ahnn J., Shim Y.H. (2006). Functional and phenotypic relevance of differentially expressed proteins in calcineurin mutants of *Caenorhabditis elegans*. Proteomics.

[33] Narula J., Pandey P., Arbustini E., Haider N., Narula N., Kolodgie F.D., dal Bello B., Semigran M.J., Bielsa-Masdeu A., Dec G.W. (1999). Apoptosis in heart failure: Release of cytochrome c from mitochondria and activation of caspase-3 in human cardiomyopathy. Proc. Natl. Acad. Sci. USA.

[34] Van Empel V.P., Bertrand A.T., Hofstra L., Crijns H.J., Doevendans P.A., de Windt L.J. (2005). Myocyte apoptosis in heart failure. Cardiovasc. Res..

[35] Nadal-Ginard B., Kajstura J., Anversa P., Leri A. (2003). A matter of life and death: Cardiac myocyte apoptosis and regeneration. J. Clin. Investig..

[36] Bishopric N.H., Andreka P., Slepak T., Webster K.A. (2001). Molecular mechanisms of apoptosis in the cardiac myocyte. Curr. Opin. Pharmacol..

[37] Haupt S., Berger M., Goldberg Z., Haupt Y. (2003). Apoptosis—The p53 network. J. Cell Sci..

[38] Tanaka M., Ito H., Adachi S., Akimoto H., Nishikawa T., Kasajima T., Marumo F., Hiroe M. (1994). Hypoxia induces apoptosis with enhanced expression of Fas antigen messenger RNA in cultured neonatal rat cardiomyocytes. Circ. Res..

[39] Hengartner M.O. (2000). The biochemistry of apoptosis. Nature.

[40] Bang S., Jeong E.J., Kim I.K., Jung Y.K., Kim K.S. (2000). Fas- and tumor necrosis factor-mediated apoptosis uses the same binding surface of FADD to trigger signal transduction. A typical model for convergent signal transduction. J. Biol. Chem..

[41] Barnhart B.C., Alappat E.C., Peter M.E. (2003). The CD95 type I/type II model. Semin. Immunol..

[42] Jeremias I., Stahnke K., Debatin K.M. (2005). CD95/Apo-1/Fas: Independent cell death induced by doxorubicin in normal cultured cardiomyocytes. Cancer Immunol. Immunother..

[43] Wang Y., Wu Y., Luo K., Liu Y., Zhou M., Yan S., Shi H., Cai Y. (2013). The protective effects of selenium on cadmium-induced oxidative stress and apoptosis via mitochondria pathway in mice kidney. Food Chem. Toxicol..

[44] Zamzami N., Kroemer G. (2001). The mitochondrion in apoptosis: How Pandora’s box opens. Nat. Rev. Mol. Cell Biol..

[45] Ryan K.M., Phillips A.C., Vousden K.H. (2001). Regulation and function of the p53 tumor suppressor protein. Curr. Opin. Cell Biol..

[46] Lee S.D., Chu C.H., Huang E.J., Lu M.C., Liu J.Y., Liu C.J., Hsu H.H., Lin J.A., Kuo W.W., Huang C.Y. (2006). Roles of insulin-like growth factor II in cardiomyoblast apoptosis and in hypertensive rat heart with abdominal aorta ligation. Am. J. Physiol. Endocrinol. Metab..

[47] Kuo W.W., Chu C.Y., Wu C.H., Lin J.A., Liu J.Y., Hsieh Y.H., Ueng K.C., Lee S.D., Hsieh D.J., Hsu H.H. (2005). Impaired IGF-I signalling of hypertrophic hearts in the developmental phase of hypertension in genetically hypertensive rats. Cell Biochem. Funct..

[48] Sun H.Y., Zhao R.R., Zhi J.M. (2000). Insulin-like growth factor I inhibits cardiomyocyte apoptosis and the underlying signal transduction pathways. Methods Find. Exp. Clin. Pharmacol..

[49] Parrizas M., Saltiel A.R., LeRoith D. (1997). Insulin-like growth factor 1 inhibits apoptosis using the phosphatidylinositol 3′-kinase and mitogen-activated protein kinase pathways. J. Biol. Chem..

[50] Matsui T., Davidoff A.J. (2007). Assessment of PI-3 kinase and Akt in ischemic heart diseases in diabetes. Methods Mol. Med..

[51] Aikawa R., Nawano M., Gu Y., Katagiri H., Asano T., Zhu W., Nagai R., Komuro I. (2000). Insulin prevents cardiomyocytes from oxidative stress-induced apoptosis through activation of PI3 kinase/Akt. Circulation.

[52] Datta S.R., Brunet A., Greenberg M.E. (1999). Cellular survival: A play in three Akts. Genes Dev..

[53] Yamamura T., Otani H., Nakao Y., Hattori R., Osako M., Imamura H. (2001). IGF-I differentially regulates Bcl-xL and Bax and confers myocardial protection in the rat heart. Am. J. Physiol. Heart Circ. Physiol..

[54] Haigis M.C., Sinclair D.A. (2010). Mammalian sirtuins: Biological insights and disease relevance. Annu. Rev. Pathol..

[55] Guarente L. (2013). Introduction: Sirtuins in aging and diseases. Methods Mol. Biol..

[56] Verdin E., Hirschey M.D., Finley L.W., Haigis M.C. (2010). Sirtuin regulation of mitochondria: Energy production, apoptosis, and signaling. Trends Biochem. Sci..

[57] Cohen H.Y., Miller C., Bitterman K.J., Wall N.R., Hekking B., Kessler B., Howitz K.T., Gorospe M., de Cabo R., Sinclair D.A. (2004). Calorie restriction promotes mammalian cell survival by inducing the SIRT1 deacetylase. Science.

[58] De Kreutzenberg S.V., Ceolotto G., Papparella I., Bortoluzzi A., Semplicini A., Dalla Man C., Cobelli C., Fadini G.P., Avogaro A. (2010). Downregulation of the longevity-associated protein sirtuin 1 in insulin resistance and metabolic syndrome: Potential biochemical mechanisms. Diabetes.

[59] Ma L., Li Y. (2015). SIRT1: Role in cardiovascular biology. Clin. Chim. Acta.

[60] Shen T., Ding L., Ruan Y., Qin W., Lin Y., Xi C., Lu Y., Dou L., Zhu Y., Cao Y. (2014). SIRT1 functions as an important regulator of estrogen-mediated cardiomyocyte protection in angiotensin II-induced heart hypertrophy. Oxid. Med. Cell. Longev..

[61] Alcendor R.R., Gao S., Zhai P., Zablocki D., Holle E., Yu X., Tian B., Wagner T., Vatner S.F., Sadoshima J. (2007). SIRT1 regulates aging and resistance to oxidative stress in the heart. Circ. Res..

[62] Shi G.F., An L.J., Jiang B., Guan S., Bao Y.M. (2006). Alpinia protocatechuic acid protects against oxidative damage *in vitro* and reduces oxidative stress *in vivo*. Neurosci. Lett..

[63] An L.J., Guan S., Shi G.F., Bao Y.M., Duan Y.L., Jiang B. (2006). Protocatechuic acid from *Alpinia oxyphylla* against MPP^+^-induced neurotoxicity in PC12 cells. Food Chem. Toxicol..

[64] Chang Y.M., Tsai C.T., Wang C.C., Chen Y.S., Lin Y.M., Kuo C.H., Tzang B.S., Chen R.J., Tsai F.J., Huang C.Y. (2013). Alpinate oxyphyllae fructus (*Alpinia Oxyphylla* MIQ) extracts inhibit angiotensin-II induced cardiac apoptosis in H9c2 cardiomyoblast cells. Biosci. Biotechnol. Biochem..

[65] Koo B.S., Lee W.C., Chang Y.C., Kim C.H. (2004). Protective effects of alpinae oxyphyllae fructus (*Alpinia oxyphylla* MIQ) water-extracts on neurons from ischemic damage and neuronal cell toxicity. Phytother. Res..

[66] Shui G., Bao Y.M., Bo J., An L.J. (2006). Protective effect of protocatechuic acid from *Alpinia oxyphylla* on hydrogen peroxide-induced oxidative PC12 cell death. Eur. J. Pharmacol..

[67] Yu X., An L., Wang Y., Zhao H., Gao C. (2003). Neuroprotective effect of *Alpinia oxyphylla* Miq. fruits against glutamate-induced apoptosis in cortical neurons. Toxicol. Lett..

[68] Shoji N., Umeyama A., Takemoto T., Ohizumi Y. (1984). Isolation of a cardiotonic principle from *Alpinia oxyphylla*. Planta Med..

[69] Lai C.H., Ho T.J., Kuo W.W., Day C.H., Pai P.Y., Chung L.C., Liao P.H., Lin F.H., Wu E.T., Huang C.Y. (2014). Exercise training enhanced SIRT1 longevity signaling replaces the IGF1 survival pathway to attenuate aging-induced rat heart apoptosis. Age.

[70] Cheng S.M., Ho T.J., Yang A.L., Chen I.J., Kao C.L., Wu F.N., Lin J.A., Kuo C.H., Ou H.C., Huang C.Y. (2013). Exercise training enhances cardiac IGFI-R/PI3K/Akt and Bcl-2 family associated pro-survival pathways in streptozotocin-induced diabetic rats. Int. J. Cardiol..

[71] Kuo W.W., Wu C.H., Lee S.D., Lin J.A., Chu C.Y., Hwang J.M., Ueng K.C., Chang M.H., Yeh Y.L., Wang C.J. (2005). Second-hand smoke-induced cardiac fibrosis is related to the Fas death receptor apoptotic pathway without mitochondria-dependent pathway involvement in rats. Environ. Health Perspect..

[72] Lin P.P., Hsieh Y.M., Kuo W.W., Lin Y.M., Yeh Y.L., Lin C.C., Tsai F.J., Tsai C.H., Huang C.Y., Tsai C.C. (2013). Probiotic-fermented purple sweet potato yogurt activates compensatory IGFIR/PI3K/Akt survival pathways and attenuates cardiac apoptosis in the hearts of spontaneously hypertensive rats. Int. J. Mol. Med..

[73] Van Tol B.A., Huijsmans R.J., Kroon D.W., Schothorst M., Kwakkel G. (2006). Effects of exercise training on cardiac performance, exercise capacity and quality of life in patients with heart failure: A meta-analysis. Eur. J. Heart Fail..

[74] Huang C.Y., Yang A.L., Lin Y.M., Wu F.N., Lin J.A., Chan Y.S., Tsai F.J., Tsai C.H., Kuo C.H., Lee S.D. (2012). Anti-apoptotic and pro-survival effects of exercise training on hypertensive hearts. J. Appl. Physiol..

[75] Kubli D.A., Gustafsson A.B. (2012). Mitochondria and mitophagy: The yin and yang of cell death control. Circ. Res..

[76] Go A.S., Mozaffarian D., Roger V.L., Benjamin E.J., Berry J.D., Blaha M.J., Dai S., Ford E.S., Fox C.S., Franco S. (2014). Executive summary: Heart disease and stroke statistics—2014 Update: A report from the American Heart Association. Circulation.

[77] Hausenloy D.J., Yellon D.M. (2013). Myocardial ischemia-reperfusion injury: A neglected therapeutic target. J. Clin. Investig..

[78] Maack C., Kartes T., Kilter H., Schafers H.J., Nickenig G., Bohm M., Laufs U. (2003). Oxygen free radical release in human failing myocardium is associated with increased activity of Rac1-GTPase and represents a target for statin treatment. Circulation.

[79] Nickel A.G., von Hardenberg A., Hohl M., Loffler J.R., Kohlhaas M., Becker J., Reil J.C., Kazakov A., Bonnekoh J., Stadelmaier M. (2015). Reversal of mitochondrial transhydrogenase causes oxidative stress in heart failure. Cell Metab..

[80] Ho S.C., Liu J.H., Wu R.Y. (2003). Establishment of the mimetic aging effect in mice caused by d-galactose. Biogerontology.

[81] Deng H.B., Cheng C.L., Cui D.P., Li D.D., Cui L., Cai N.S. (2006). Structural and functional changes of immune system in aging mouse induced by d-galactose. Biomed. Environ. Sci..

[82] Salganik R.I., Solovyova N.A., Dikalov S.I., Grishaeva O.N., Semenova L.A., Popovsky A.V. (1994). Inherited enhancement of hydroxyl radical generation and lipid peroxidation in the S strain rats results in DNA rearrangements, degenerative diseases, and premature aging. Biochem. Biophys. Res. Commun..

[83] Bosch A.M. (2006). Classical galactosaemia revisited. J. Inherit. Metab. Dis..

[84] Hao L., Huang H., Gao J., Marshall C., Chen Y., Xiao M. (2014). The influence of gender, age and treatment time on brain oxidative stress and memory impairment induced by d-galactose in mice. Neurosci. Lett..

[85] Aso Y., Inukai T., Tayama K., Takemura Y. (2000). Serum concentrations of advanced glycation endproducts are associated with the development of atherosclerosis as well as diabetic microangiopathy in patients with type 2 diabetes. Acta Diabetol..

[86] Cui X., Wang L., Zuo P., Han Z., Fang Z., Li W., Liu J. (2004). d-Galactose-caused life shortening in *Drosophila melanogaster* and *Musca domestica* is associated with oxidative stress. Biogerontology.

[87] Dias N., Bailly C. (2005). Drugs targeting mitochondrial functions to control tumor cell growth. Biochem. Pharmacol..

[88] Schulze-Osthoff K., Ferrari D., Los M., Wesselborg S., Peter M.E. (1998). Apoptosis signaling by death receptors. Eur. J. Biochem..

[89] Rao R.V., Ellerby H.M., Bredesen D.E. (2004). Coupling endoplasmic reticulum stress to the cell death program. Cell Death Differ..

[90] De la Fuente M. (2002). Effects of antioxidants on immune system ageing. Eur. J. Clin. Nutr..

[91] Mann D.L. (2015). Innate immunity and the failing heart: The cytokine hypothesis revisited. Circ. Res..

[92] Karin M., Lin A. (2002). NF-κB at the crossroads of life and death. Nat. Immunol..

[93] Schmidt K.N., Amstad P., Cerutti P., Baeuerle P.A. (1995). The roles of hydrogen peroxide and superoxide as messengers in the activation of transcription factor NF-κB. Chem. Biol..

[94] Oeckinghaus A., Ghosh S. (2009). The NF-κB family of transcription factors and its regulation. Cold Spring Harb. Perspect. Biol..

[95] Kauppinen A., Suuronen T., Ojala J., Kaarniranta K., Salminen A. (2013). Antagonistic crosstalk between NF-κB and SIRT1 in the regulation of inflammation and metabolic disorders. Cell Signal..

[96] Ciou S.Y., Hsu C.C., Kuo Y.H., Chao C.Y. (2014). Effect of wild bitter gourd treatment on inflammatory responses in BALB/c mice with sepsis. BioMedicine.

[97] Kawai T., Akira S. (2006). TLR signaling. Cell Death Differ..

[98] Inoue M., Shinohara M.L. (2013). NLRP3 Inflammasome and MS/EAE. Autoimmune Dis..

[99] Ito M., Shichita T., Okada M., Komine R., Noguchi Y., Yoshimura A., Morita R. (2015). Bruton’s tyrosine kinase is essential for NLRP3 inflammasome activation and contributes to ischaemic brain injury. Nat. Commun..

[100] Zhong Y., Kinio A., Saleh M. (2013). Functions of NOD-like receptors in human diseases. Front. Immunol..

[101] Coggins M., Rosenzweig A. (2012). The fire within: Cardiac inflammatory signaling in health and disease. Circ. Res..

[102] Matsushima S., Sadoshima J. (2015). The role of sirtuins in cardiac disease. Am. J. Physiol. Heart Circ. Physiol..

[103] Yeung F., Hoberg J.E., Ramsey C.S., Keller M.D., Jones D.R., Frye R.A., Mayo M.W. (2004). Modulation of NF-κB-dependent transcription and cell survival by the SIRT1 deacetylase. EMBO J..

[104] Palomer X., Alvarez-Guardia D., Rodriguez-Calvo R., Coll T., Laguna J.C., Davidson M.M., Chan T.O., Feldman A.M., Vazquez-Carrera M. (2009). TNF-α reduces PGC-1α expression through NF-κB and p38 MAPK leading to increased glucose oxidation in a human cardiac cell model. Cardiovasc. Res..

[105] Lee G.S., Subramanian N., Kim A.I., Aksentijevich I., Goldbach-Mansky R., Sacks D.B., Germain R.N., Kastner D.L., Chae J.J. (2012). The calcium-sensing receptor regulates the NLRP3 inflammasome through Ca^2+^ and cAMP. Nature.

[106] Rossol M., Pierer M., Raulien N., Quandt D., Meusch U., Rothe K., Schubert K., Schoneberg T., Schaefer M., Krugel U. (2012). Extracellular Ca^2+^ is a danger signal activating the NLRP3 inflammasome through G protein-coupled calcium sensing receptors. Nat. Commun..

[107] Qu Y., Misaghi S., Izrael-Tomasevic A., Newton K., Gilmour L.L., Lamkanfi M., Louie S., Kayagaki N., Liu J., Komuves L. (2012). Phosphorylation of NLRC4 is critical for inflammasome activation. Nature.

[108] Kotas M.E., Gorecki M.C., Gillum M.P. (2013). Sirtuin-1 is a nutrient-dependent modulator of inflammation. Adipocyte.

[109] Fuentes-Antras J., Ioan A.M., Tunon J., Egido J., Lorenzo O. (2014). Activation of toll-like receptors and inflammasome complexes in the diabetic cardiomyopathy-associated inflammation. Int. J. Endocrinol..

[110] Sudhir R., Sukhodub A., Du Q., Jovanovic S., Jovanovic A. (2011). Ageing-induced decline in physical endurance in mice is associated with decrease in cardiac SUR2A and increase in cardiac susceptibility to metabolic stress: Therapeutic prospects for up-regulation of SUR2A. Biogerontology.

[111] Du Q., Jovanovic S., Sukhodub A., Jovanovic A. (2010). Infection with AV-SUR2A protects H9c2 cells against metabolic stress: A mechanism of SUR2A-mediated cytoprotection independent from the K_ATP_ channel activity. Biochim. Biophys. Acta.

[112] Du Q., Jovanovic S., Clelland A., Sukhodub A., Budas G., Phelan K., Murray-Tait V., Malone L., Jovanovic A. (2006). Overexpression of SUR2A generates a cardiac phenotype resistant to ischemia. FASEB J..

[113] Mohammed Abdul K.S., Jovanovic S., Du Q., Sukhodub A., Jovanovic A. (2015). Mild hypoxia *in vivo* regulates cardioprotective SUR2A: A role for Akt and LDH. Biochim. Biophys. Acta.

[114] Mohammed Abdul K.S., Jovanovic S., Du Q., Sukhodub A., Jovanovic A. (2015). A link between ATP and SUR2A: A novel mechanism explaining cardioprotection at high altitude. Int. J. Cardiol..

[115] Crawford R.M., Jovanovic S., Budas G.R., Davies A.M., Lad H., Wenger R.H., Robertson K.A., Roy D.J., Ranki H.J., Jovanovic A. (2003). Chronic mild hypoxia protects heart-derived H9c2 cells against acute hypoxia/reoxygenation by regulating expression of the SUR2A subunit of the ATP-sensitive K^+^ channel. J. Biol. Chem..

[116] Sukhodub A., Du Q., Jovanovic S., Jovanovic A. (2010). Nicotinamide-rich diet protects the heart against ischaemia-reperfusion in mice: A crucial role for cardiac SUR2A. Pharmacol. Res..

[117] Mohammed Abdul K.S., Jovanovic S., Sukhodub A., Du Q., Jovanovic A. (2014). Upregulation of cardioprotective SUR2A by sub-hypoxic drop in oxygen. Biochim. Biophys. Acta.

[118] Poljsak B., Milisav I. (2016). The NAD^+^-depletion theory of ageing: NAD^+^ as the link between oxidative stress, inflammation, caloric restriction, exercise, DNA repair, longevity and health span. Rejuvenation Res..

[119] Imai S., Armstrong C.M., Kaeberlein M., Guarente L. (2000). Transcriptional silencing and longevity protein SIR2 is an NAD-dependent histone deacetylase. Nature.

[120] Canto C., Houtkooper R.H., Pirinen E., Youn D.Y., Oosterveer M.H., Cen Y., Fernandez-Marcos P.J., Yamamoto H., Andreux P.A., Cettour-Rose P. (2012). The NAD^+^ precursor nicotinamide riboside enhances oxidative metabolism and protects against high-fat diet-induced obesity. Cell Metab..

[121] Lin S.J., Ford E., Haigis M., Liszt G., Guarente L. (2004). Calorie restriction extends yeast life span by lowering the level of NADH. Genes Dev..

